# COVID-19-related retinal microvasculopathy and systemic implications in patients with severe disease: results from the Methuselah study

**DOI:** 10.3389/fmed.2024.1294432

**Published:** 2024-01-26

**Authors:** Niccolò Castellino, Antonio Longo, Andrea Russo, Vincenza Bonfiglio, Matteo Fallico, Mario Damiano Toro, Francesco Cappellani, Marco Grillo, Agostino Gaudio, Lorenzo Lo Cicero, Concetto Sessa, Michele Colaci, Lorenzo Malatino, Pietro Castellino, Teresio Avitabile, Luca Zanoli

**Affiliations:** ^1^Department of Ophthalmology, University of Catania, Catania, Italy; ^2^Department of Experimental Biomedicine and Clinical Neuroscience, University of Palermo, Palermo, Italy; ^3^Department of General Ophthalmology, Medical University of Lublin, Lublin, Poland; ^4^Department of Clinical and Experimental Medicine, University of Catania, Catania, Italy

**Keywords:** macula, optical coherence tomography angiography, vascular density, foveal avascular zone, acute kidney injury, arterial stiffness, inflammation, pulse wave velocity

## Abstract

**Objectives:**

To assess the reversibility of retinal microvascular changes in the long term and to investigate the potential links with other vascular diseases of COVID-19.

**Methods:**

We designed a prospective multicenter observational study. Patients were enrolled from the Methuselah study cohort. Retinal vascular function was studied in these patients using optical coherence tomography angiography (OCTA); aortic stiffness was measured using aortic pulse wave velocity. These examinations were performed 1 (Visit 1) and 12 (Visit 2) months after the hospital discharge for severe COVID-19. A control subject group matched for age and sex was included to define normal values.

**Results:**

A total of 28 control subjects (56 eyes) and 25 patients (50 eyes) completed the scheduled OCTA assessment; 18 patients (36 eyes) also completed the macrovascular examination. Compared to controls, the vessel density of the superficial capillary plexus (SCP) was reduced, whereas the foveal avascular zone area was enlarged at Visit 1 (*p* = 0.016 and < 0.001, respectively) and was not modified after the 12-month follow-up in COVID-19 patients (*p* = 0.011 and 0.001, respectively). Higher inflammation and lower renal function during hospitalization were linked to higher aortic stiffness and reduced vessel density of the SCP 1 month after the acute phase of COVID-19. A slower recovery of aortic dysfunction was linked to worse retinal vascular outcomes at Visit 2.

**Conclusion:**

Retinal vascular alterations were not reversible 12 months after COVID-19 and were linked to inflammation and renal dysfunction during hospitalization as well as to aortic stiffness measured during follow-up.

## Introduction

Coronavirus disease-19 (COVID-19) was a global pandemic caused by severe acute respiratory syndrome coronavirus 2 (SARS-CoV-2). It primarily infects the lungs and airway cells by binding the ACE-2 receptor, which is expressed in several cellular populations including endothelial cells (1, 2). However, many aspects of the pathogenesis of the vascular disease related to COVID-19, including the involvement of the microvascular retinal bed and the potential interplay with inflammation and macrovascular beds, are still not fully elucidated.

Optical coherence tomography angiography (OCTA) is a retinal imaging technique that allows the assessment of the retina and the neovascular network in a non-invasive fashion in patients affected by systemic diseases (3). The OCTA investigation of the retina micro-vasculature showed high accuracy in detecting retinal vascular alterations in other models of inflammation (4–7).

During the acute phase of COVID-19 and a few months after its resolution, several cross-sectional studies employing OCTA showed COVID-related damage to the vascular retinal bed, which varied according to the severity of the disease (8, 9). However, few data are available on the evolution of these findings over time. Thus, mid- and long-term effects of COVID-19 on retinal vascular changes need to be further investigated (10).

To assess whether the retinal vascular changes in patients with recent severe COVID-19 are still evident or reversible in the long term, we designed a multicenter observational prospective study. Moreover, we also aimed to better understand the further involvement of microvascular and macrovascular beds in this setting.

## Materials and methods

### Study 1

**Figure 1:**
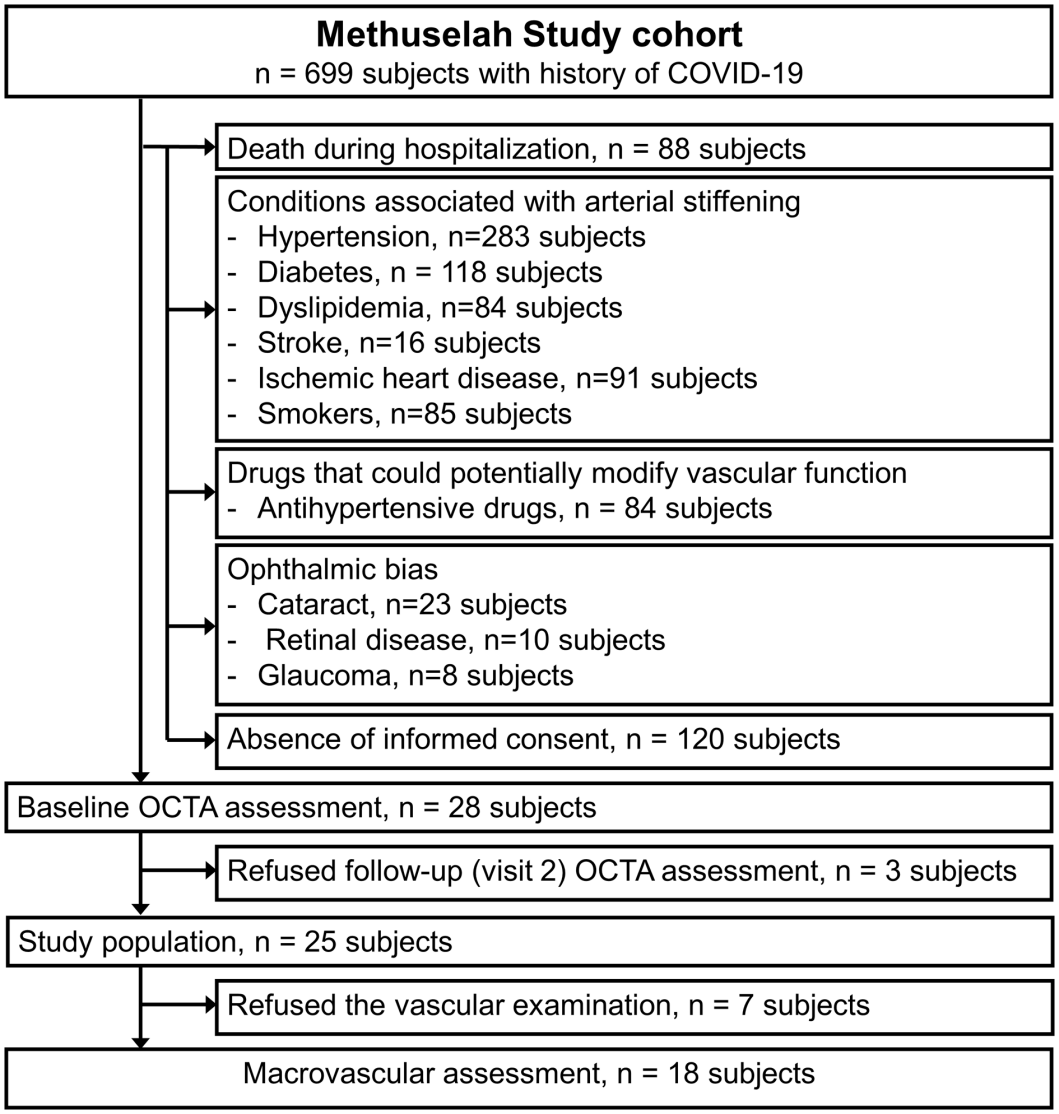
A flowchart describing the selection of subjects for the present analysis.

Study 1 was a multicenter observational longitudinal study. Overall, a total of 28 patients (12 women) of Italian ethnicity with a previous hospitalization for COVID-19 from September 2020 to April 2021 in three separate COVID-19 units from three different hospitals in Italy (Infectious Diseases, Policlinico di Catania, Catania; Internal Medicine, Cannizzaro Hospital, Catania; and San Marco Hospital, Catania) were included in this analysis (Figure 1). Patients included in the present study were enrolled from the Methuselah study cohort; the aortic stiffness assays and the renal function assessments from this study partially overlap with our previous analysis (11). The patient group was paired with a control group of 28 subjects of the same ethnicity, who were matched for age and sex, recruited from the Department of Ophthalmology of the University of Catania and without any history of ocular disease. The exclusion criteria were as follows: previous ocular inflammation, glaucoma, anamnesis of ocular trauma or surgery, high myopia ≥6.0 diopters, hyperopia ≥3.0 diopters, amblyopia, opacity of dioptric media, retinal and optic disk clinical abnormalities, hypertension, diabetes, and any ocular or systemic condition, which could bias OCTA measurements. Moreover, patients with COVID-19 and controls with conditions associated with arterial stiffening (diabetes, estimated glomerular filtration rate (eGFR) <60 mL/min/1.73 m^2^, dyslipidemia, stroke, ischemic heart disease, and current or former smokers [i.e., smoking cessation >1 year from hospitalization]) and those taking prescription medication (drugs), which could potentially modify vascular function (antihypertensive drugs), were also excluded from this analysis.

COVID-19 status was confirmed using a polymerase chain reaction. All patients in Study 1 were hospitalized for COVID-19. The severity of COVID-19 during hospitalization was defined according to the World Health Organization clinical progression score [WHO Working Group 2020]. Written informed consent was obtained from each participant before enrollment. The study protocol conformed to the ethical guidelines of the 1975 Declaration of Helsinki and was approved by the ethics committee on research on humans of the University of Catania. The primary outcome measure was to identify retinal vascular alterations in comparison to controls as well as changes over the follow-up period in COVID-19 subjects. Secondary outcomes included the analysis of clinical factors associated with the retinal vascular status.

### Study 2

Study 2 was a multicenter observational longitudinal study. A non-invasive examination of the aortic function was proposed for all COVID-19 patients enrolled in Study 1. A total of 18 out of 25 patients underwent vascular examination (7 refused it) after 1 and 12 months of follow-up (Visit 1 and Visit 2, respectively) and were included in Study 2. The primary outcome was to investigate the potential links between inflammation, microvascular function (the retinal vascular and glomerular beds), and macrovascular function (aortic stiffness).

### Ophthalmic examination

Patients and controls underwent an ophthalmic examination at the Ophthalmological Department of the University of Catania (Catania, Italy). All patients underwent a complete ophthalmic examination, including best-corrected visual acuity (BCVA) test, biomicroscopy of the anterior segment, fundus examination after pupil dilation with 1% tropicamide, and OCTA. Patients were examined by OCTA 1 month (Visit 1) and 12 months after hospital discharge.

OCTA of the macula was carried out by employing AngioVue XR Avanti (Optovue Inc., Fremont, California, United States). The technology of OCTA has been previously described in detail (12). The A-scan rate was 70,000 per second with a bandwidth of 50 nm and a light source centered of 840 nm. The OCTA images of the macula (6 × 6 mm) were centered on the foveola. Each volume contained 400 × 400 A-scans with two consecutive B-scans captured in each fixed position. The split-spectrum amplitude-decorrelation angiography (SSADA) algorithm was used to capture the dynamic motion of red blood cells. AngioVue software automatically segments the area into four layers, including the superficial capillary plexus layer (SCP), deep capillary plexus layer (DCP), outer retinal layer, and choriocapillaris (13). Vascular density (VD) was defined as the percentage of the tested area occupied by vessels. The VD in the superficial and deep vascular plexi were automatically calculated by Optovue software with a density function. The foveal avascular zone (FAZ) area (mm^2^) was analyzed by the software with a non-flow function.

Data on foveal thickness (FT, μm) were automatically provided by the machine simultaneously with VD data. Macular whole image vascular density values (wVD) in the SCP and in the DCP (superficial wVD and deep wVD, respectively) were calculated on the entire field of 6 × 6 mm scans centered on the fovea. In addition, the analyzes of the vascular density were focused on the sub-sectors of the whole image, including the foveal (superficial fVD and deep fVD, respectively) and parafoveal (superficial pVD and deep pVD, respectively) areas.

OCTA of the optic nerve head (ONH) was carried out with a scan field of 4.5 × 4.5 mm centered on the optic disk. The vessel density of the radial peripapillary capillary (RPC) layer was analyzed by AngioVue software.

### Vascular examination protocol

Vascular examination was performed as previously described (11). Briefly, all participants included in Study 2 were studied between 09:00 a.m. and 11:00 a.m. while fasting in a centralized vascular laboratory by an expert operator (L.Z.) blinded to clinical data and in a quiet room with a controlled temperature of 22 ± 1°C after 15 min of rest in a supine position. Aortic pulse wave velocity (aPWV), a measure of aortic stiffness, was measured with a SphygmoCor device (SphygmoCor system, AtCorMedical, Sydney, Australia), as previously reported (14), using the foot-to-foot velocity method, the intersecting tangent algorithm, and the direct distance between the measurement sites (15): aPWV (m/s) = 0.8 × (carotid-femoral direct distance [m]/Δt). The mean value of two consecutive recordings was used for this analysis. When the difference between the two measurements was ≥0.5 m/s, a third recording was performed, and the median value was used. Vascular examination was performed at 1 and 12 months after the acute phase of COVID-19 (Visit 1 and Visit 2, respectively).

### Clinical variables

Enzyme immunoassays were used for the quantitative determination of high-sensitivity C-reactive protein (hs-CRP). The estimated glomerular filtration rate (eGFR) was estimated using the Chronic Kidney Disease Epidemiology Collaboration formula (16). Higher hs-CRP and lower eGFR during hospitalization for COVID-19 as well as the slope of eGFR from hospitalization to Visit 1 were used in this analysis. In patients with COVID-19, clinical data were collected within 1 week of Visit 1 and Visit 2. Molecular tests for SARS-CoV-2 RNA were performed using a reverse-transcription polymerase chain reaction assay.

### Statistical analyzes

Continuous variables are presented as means (±standard deviation); categorical variables are presented as percentages. A comparison of continuous variable values among the groups at different time points was performed by a one-way analysis of variance (ANOVA). Significant comparisons of a one-way ANOVA were tested using post-hoc analysis with Tukey’s HSD test.

A partial least squares structural equation modeling (PLS-SEM) was performed as previously reported (11). Briefly, PLS-SEM was used to perform mediation analyzes and explore the potential links between inflammation, renal function, and vascular dysfunction in patients with COVID-19 (17). Based on the mediation analysis, we quantified both the direct and indirect (mediated) effects of aPWV on the SCP. A mediation effect was confirmed when (a) exposure (i.e., eGFR during hospitalization) was significantly correlated with the mediator (i.e., aPWV) and (b) the mediator was significantly correlated with the outcome (i.e., SCP). All statistical analyzes were performed using SPSS (version 22, IBM, New York, United States) except for PLS-SEM analyzes that were performed using SmartPLS 4 software (SmartPLS GmbH, Boenningstedt, Germany). A two-tailed value of p of <0.05 was considered to indicate statistical significance.

## Results

### Study 1

#### Clinical characteristics of patients with COVID-19

Three patients (six eyes) of the COVID-19 group were ruled out after V1 examination because of consensus denied over the follow-up. Therefore, a total of 50 eyes of 25 patients (14 men and 11 women) hospitalized for COVID-19 and 56 eyes of control subjects (14 men and 14 women) performed both the visits and were included in Study 1. The age was comparable between the COVID-19 and the control group (52.6 ± 14.2 vs. 49.2 ± 14.4 years, respectively, *p* = 0.452). Accordingly, no significant differences in axial length, BCVA, and IOP values were found between the two groups. Complete demographic and clinical data are listed in Table 1.

**Table 1:** Demographic and clinical characteristics of patients affected by COVID-19 and control subjects.

	COVID (*n* = 25)	Controls (*n* = 28)	Level of statistical significance (*p* < 0.05)
Age (years), mean ± SD	52.6 ± 14.2	49.2 ± 14.4	0.542
Gender (man:women)	14:11	14:14	–
O_2_ therapy during hospitalization			
- low-flux O_2_, %	48	–	–
- high-flux O_2_/CPAP, %	52	–	–
*Ophthalmic characteristics*			
BCVA (logMAR)	0.03 ± 0.07	0.02 ± 0.04	0.436
Axial length, (mm), mean ± SD	24.3 ± 1	24.1 ± 0.9	0.530
IOP (mmhg), mean ± SD	14.4 ± 2.6	15 ± 2.8	0.426

SD, standard deviation; O_2_, oxygen; BCVA, best-corrected visual acuity; D, diopters; IOP, intraocular pressure; *n*, number.

#### OCTA analysis

At OCTA analysis, no significant difference was found between Visit 1 and Visit 2 in patients hospitalized for COVID-19. Complete OCTA data are listed in Table 2. The whole SCP vessel density at Visit 1 turned out to be significantly reduced compared to controls (46.0 ± 7.3 and 48.7 ± 3.0, respectively, *p* = 0.016). By contrast, no significant difference was detected in the DCP.

**Table 2:** OCTA data in patients hospitalized for COVID-19 disease and control subjects.

	COVID		CONTROLS	*p* (ANOVA)	*A* vs. B*	*A* vs. C*	*B* vs. C*
	Visit 1 A	Visit 2 B	Baseline C				
FAZ (mm^2^), mean ± SD	0.27 ± 0.10	0.27 ± 0.09	0.21 ± 0.08	<0.001	0.985	<0.001	0.001
FT (μm), mean ± SD	261 ± 39	258 ± 25	264 ± 19	0.649	–	–	–
Superficial wVD (%), mean ± SD	46.0 ± 7.3	45.9 ± 3.4	48.7 ± 3.0	0.005	0.992	0.016	0.011
Superficial fVD (%), mean ± SD	20.2 ± 8.2	20.2 ± 6.1	24.5 ± 6.7	0.001	0.999	0.005	0.006
Superficial pVD (%), mean ± SD	47.8 ± 5.8	46.9 ± 3.9	49.6 ± 4.2	0.015	0.653	0.126	0.013
Superficial periVD (%), mean ± SD	47.3 ± 4.3	46.2 ± 3.4	49.4 ± 3.0	<0.001	0.279	0.009	<0.001
Deep wVD (%), mean ± SD	46.9 ± 5.7	45.9 ± 4.1	47.4 ± 5.3	0.303	–	–	–
Deep fVD (%), mean ± SD	36.5 ± 7.8	37.1 ± 6.5	41.1 ± 6.3	0.001	0.897	0.003	0.011
Deep pVD (%), mean ± SD	51.5 ± 5.0	50.9 ± 3.9	52.3 ± 4.5	0.283	–	–	–
Deep periVD (%), mean ± SD	47.8 ± 6.3	47.3 ± 5.5	48.3 ± 5.8	0.683	–	–	–
RCP whole VD (%), mean ± SD	48.7 ± 2.8	48.4 ± 3.6	49.5 ± 2.2	0.125	–	–	–
Inside disk VD (%), mean ± SD	48.1 ± 4.5	47.6 ± 4.2	49.7 ± 5.8	0.063	–	–	–
Peripapillary VD (%), mean ± SD	52.0 ± 3.2	52.3 ± 3.1	51.9 ± 2.5	0.813	–	–	–

FT, foveal thickness; FAZ, foveal avascular zone; VD, vascular density; SD, standard deviation; FAZ, foveal avascular zone; wVD, whole-image vascular density; fVD, foveal vascular density; pVD, parafoveal vascular density; periVD, perifoveal vascular density; RCP, radial capillary peripapillary.

^*^ Tukey’s HSD test.

Fovea SCP and DCP vessel density at Visit 1 was significantly lower in COVID-19 patients than in control subjects (*p* = 0.005 and 0.003, respectively).

Accordingly, even the fovea vessel density at the 12-month visit resulted in a reduction in both SCP and DCP in comparison with control subjects (*p* = 0.006 and 0.011, respectively). In addition, a reduction of the VD in the SCP was detected in the whole tested area in the parafovea as well as in the perifovea sectors (*p* = 0.011, *p* = 0.013, and *p* < 0.001, respectively) (Table 2).

The FAZ area of COVID-19 patients was significantly larger both at Visit 1 and Visit 2 in comparison with controls (p < 0.001 and *p* = 0.001).

OCTA of the RCP layer showed no difference in vessel density among the three groups (Table 2).

### Study 2

**Figure 2:**
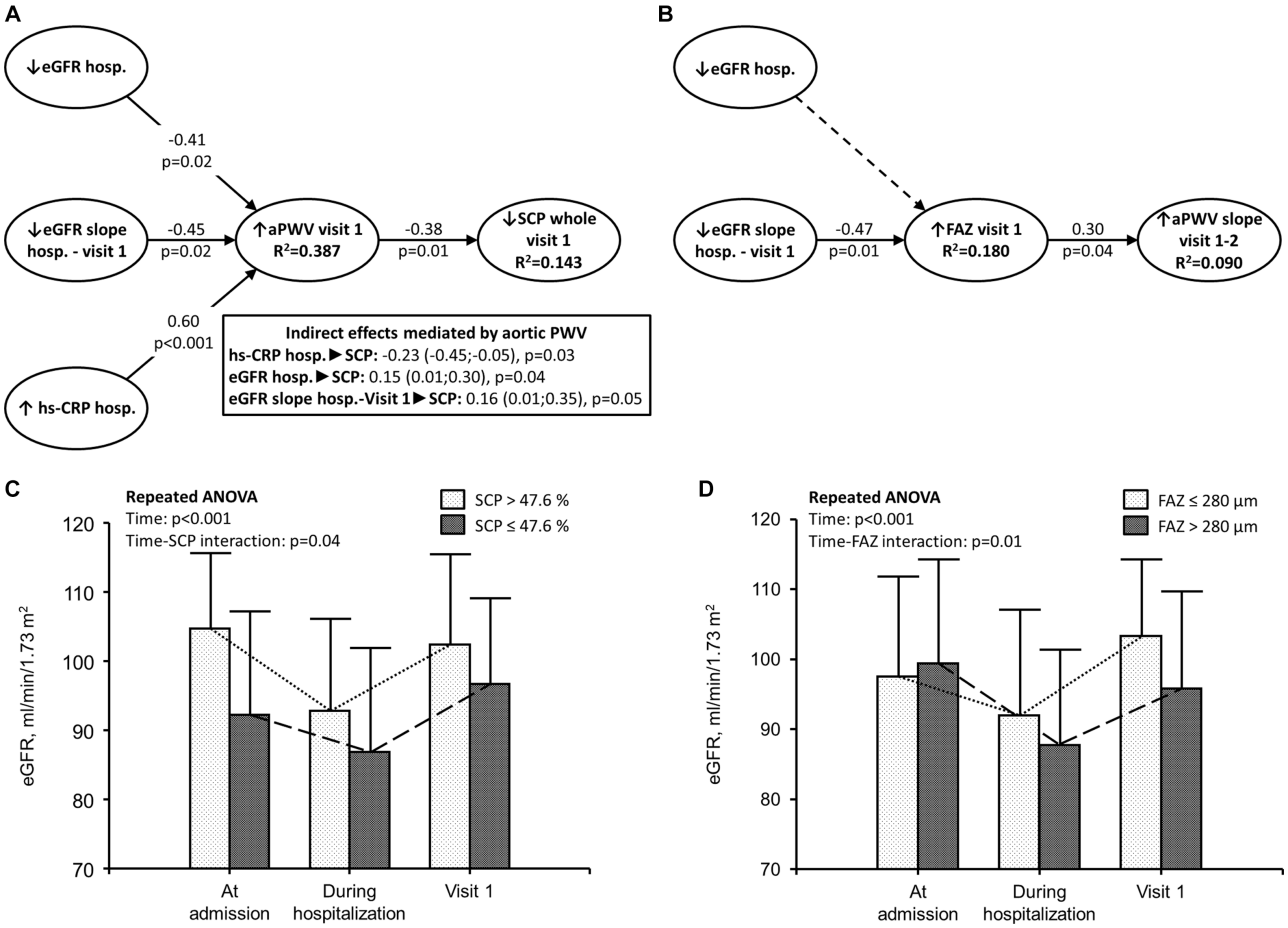
Microvascular and macrovascular alterations of COVID-19. **(A,B)** Partial least squares structural equation modeling performed in 36 eyes using a path weighting scheme. Effect of severe inflammation during the acute phase of COVID-19 on microvascular and macrovascular diseases in patients hospitalized for COVID-19. hs-CRP was measured during hospitalization for COVID-19. eGFR was calculated during hospitalization for COVID-19 and at Visit 1 (1 month after the acute phase of the disease); SCP and FAZ were measured at the time of Visit 1; aPWV was measured at Visit 1 and Visit 2 (1 and 12 months after the acute phase of the disease, respectively). **(A)** hs-CRP and eGFR measured during hospitalization as well as eGFR slope between hospitalization and Visit 1 were included as independent variables; aortic PWV at Visit 1 had a dual relationship as both an independent and a dependent variable; SCP at Visit 1 was a dependent variable. The link between the eGFR slope between hospitalization and Visit 1, FAZ at Visit 1, and aPWV slope between Visit 1 and Visit 2 is reported in panel **(B)**. Significantly direct effects are reported as continuous black lines; non-significant direct effects are reported as dotted lines. Arrows indicate the direction of the effects tested in the model. *R*^2^ indicates the variance explained by the model. For instance, in panel **(A)**, 14.3% of the variance in SCP (dependent variable) was explained by the model. **(C,D)** eGFR in patients with SCP and FAZ below and above the median value.

**Figure 3:**
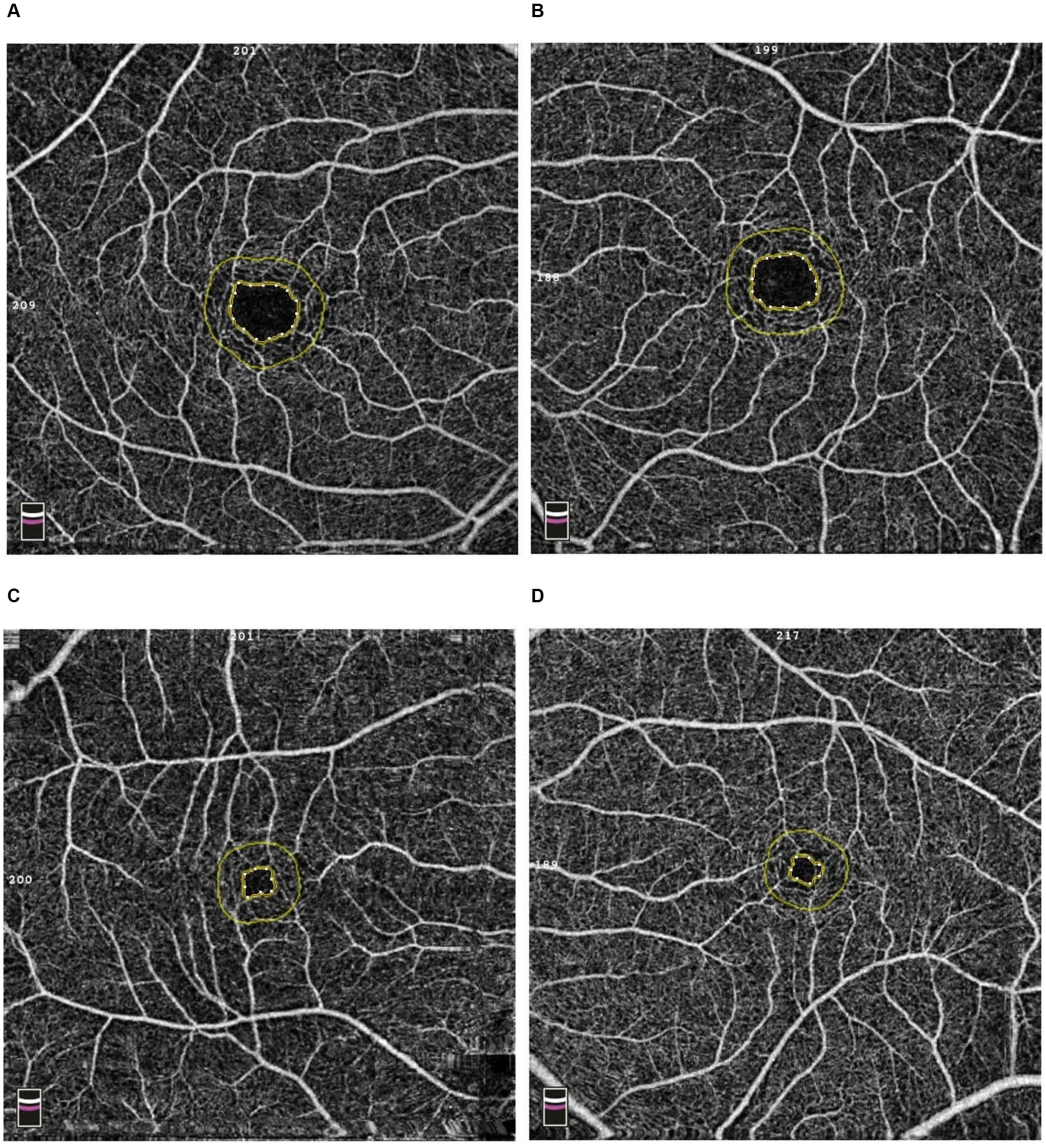
Optical coherence tomography angiography (OCTA) images of a patient with increased aortic stiffness [**(A)**: right eye; **(B)**: left eye] and without increased aortic stiffness [**(C)**: right eye; **(D)**: left eye]. The inner yellow line delimited the foveal avascular zone area that was significantly enlarged in the patient with increased aortic stiffness.

A total of 36 eyes of 18 patients (11 men and 7 women) were included in Study 2. In PLS-SEM analyzes, alterations of the glomerular, retinal, and macrovascular beds are all linked in patients with COVID-19 in the short term, 1 month after the acute phase of the disease, as both lower eGFR during hospitalization and lower improvement of eGFR from hospitalization to Visit 1 were linked to higher aPWV and lower SCP at Visit 1 (Figure 2A). Finally, microvascular (i.e., glomerular and retinal) and macrovascular (i.e., aorta) diseases of COVID-19 are linked in the long term, 12 months after the acute phase of the disease, as the lower improvement of eGFR from hospitalization to Visit 1, higher FAZ at Visit 1, and aPWV increase from Visit 1 to Visit 2 were observed (Figure 2B). OCTA analysis in one patient with increased aortic stiffness and another without increased aortic stiffness included in our study is reported in Figure 3.

## Discussion

In the present study, we observed a link between renal and retinal microvascular abnormalities with large-vessel abnormalities in both the short term and the long term in patients with severe COVID-19 disease.

The changes in the retinal vessels in patients who underwent COVID-19 infection are well known and linked to disease severity (8). Zapata et al. (18) showed a grading of vessel density alterations according to disease severity. However, limited data are available on the reversibility of these alterations over time.

We reported for the first time that the retinal changes were still evident up to 12 months after the acute phase of COVID-19 in patients hospitalized for severe disease. In particular, we found a decreased retinal vascular density in COVID-19 patients in comparison with controls in both the short term and long term (1 and 12 months after the acute phase of the disease). The superficial capillary plexus showed greater alterations than the deep capillary plexus.

In addition, we found that the degree of inflammation was associated with microvascular (i.e., eGFR reduction and retinal changes) and macrovascular diseases (i.e., aortic stiffening) 1 month after the acute phase of the disease. In more detail, an increased aortic stiffening was associated with a reduction in the vascular density at the level of the SCP. The VD of the SCP was linked to the high levels of C-reactive protein measured during the acute phase of COVID-19. These results corroborate the link between inflammation and vascular dysfunction in patients with COVID-19.

Patients who showed a decreased VD of the SCP or an enlarged FAZ, two parameters that are closely related to macular ischemia, were more often characterized by a lower reversibility of acute kidney injury (AKI; Figures 2C,D). These findings support the hypothesis that inflammation, ischemia, and vascular disease are all linked in patients with severe COVID-19. Furthermore, the improvement of eGFR 1 month after the acute phase of COVID-19 suggests that COVID-19 leads to functional and reversible acute kidney injury. On the other hand, no improvement was observed in the retinal microvascular damage over the follow-up.

Taking into account that FAZ enlargement and reduction of the SCP vascular density (19) are two biomarkers of macular ischemia (20, 21), and that patients with conditions associated with macular ischemia known before COVID-19 (i.e., those affected by diabetic retinopathy, retinal vein occlusion, and any vasculopathy) were excluded from this study, the association between the eGFR changes from hospitalization to Visit 1 and the larger FAZ area at Visit 1 (Figures 2B,D) suggest that both renal and retinal disease are caused by severe COVID-19.

Our results could suggest that the thromboinflammation previously reported during the acute phase of severe COVID-19 (22, 23) could lead to both microvascular and macrovascular diseases. In this setting, the coagulation cascade is activated, and inflammation is stimulated in a self-perpetual cycle (14, 24, 25).

One of the purposes of this study was to investigate, with a prospective design, whether the retinal capillary status, altered in the short term in COVID-19 patients, may change over longer follow-ups. In this regard, no microvascular retinal changes were observed 12 months after the hospital discharge in comparison with a 1-month visit. This finding may suggest that virus-mediated capillary closure by SARS COVID-19 produces structural changes in the retinal vascular tissues that are not reversible. However, at present, it cannot be excluded that this decline in retinal perfusion may improve over a more extended time interval from the COVID-19 infection. A more prolonged follow-up and additional studies are needed to address these questions.

We have recently reported a partial reversibility of aortic stiffening 12 months after the acute phase of the disease (11), and our results may support the hypothesis that COVID-19 leads to both functional (rapidly reversible) and structural alterations of microvascular and macrovascular beds.

The present study has several strengths. First, most of the previously published articles that have evaluated the vascular function in patients with COVID-19 were cross-sectional. The prospective design allows us to explore the causality and temporality of the potential associations. In this regard, we used PLS-SEM to test potential causal relationships between acute severe inflammation, renal function, and macrovascular and microvascular diseases in COVID-19 patients. This new powerful methodology has been successfully employed to perform mediation analyzes (26). However, larger longitudinal studies are required to confirm our results, and long-term follow-ups are needed to assess the level of reversibility of the abovementioned retinal lesions. Second, we performed a comprehensive evaluation of the vascular function in patients affected by COVID-19 and studied both microvascular and macrovascular beds. Finally, to reduce sources of bias, our study included only COVID-19 patients and control subjects without ocular or systemic conditions, which could bias OCTA measurements or clinical conditions associated with arterial stiffening.

This study has also some limitations, including a relatively small sample of eyes and the absence of a baseline ophthalmic and aortic stiffness assessment before COVID-19 as well as during the hospitalization, at the time of the peak of systemic inflammation. Finally, considering that renal function can be influenced by the patency of the renal large arteries (27), we cannot exclude that the reduced eGFR reported during hospitalization can be ascribed to a partial and reversible thrombosis of the renal arteries during the acute phase of COVID-19. In conclusion, there are extensive retinal microvascular alterations in patients affected by severe COVID-19 that are not reversible 1 year after hospital discharge and that are strictly linked with other microvascular and macrovascular changes induced by COVID-19.

## Data availability statement

The raw data supporting the conclusions of this article will be made available by the authors, without undue reservation.

## Ethics statement

The studies involving humans were approved by Comitato Etico Catania 1, Catania, Italy. The studies were conducted in accordance with the local legislation and institutional requirements. The participants provided their written informed consent to participate in this study.

## Author contributions

NC: Writing – original draft, Writing – review & editing. AL: Conceptualization, Software, Writing – review & editing. AR: Methodology, Validation, Writing – review & editing. VB: Conceptualization, Writing – review & editing. MF: Formal analysis, Investigation, Writing – review & editing. MT: Conceptualization, Data curation, Investigation, Writing – review & editing. FC: Data curation, Writing – review & editing. MG: Data curation, Writing – review & editing. AG: Conceptualization, Writing – review & editing. LC: Data curation, Writing – review & editing. CS: Methodology, Supervision, Writing – review & editing. MC: Conceptualization, Writing – review & editing. LM: Data curation, Methodology, Writing – review & editing. PC: Supervision, Writing – review & editing. TA: Investigation, Writing – review & editing. LZ: Conceptualization, Investigation, Supervision, Writing – review & editing.
